# Variation in the Physical and Functional Properties of Yam (*Dioscorea* spp.) Flour Produced by Different Processing Techniques

**DOI:** 10.3390/foods10061341

**Published:** 2021-06-10

**Authors:** Nurdi Setyawan, John Solomon Maninang, Sakae Suzuki, Yoshiharu Fujii

**Affiliations:** 1 Indonesian Center for Agricultural Postharvest Research and Development, Jl. Tentara Pelajar No.12, Kampus Penelitian Pertanian Cimanggu, Bogor 16114, West Java, Indonesia; 2 Department of International Environmental and Agricultural Science, Tokyo University of Agriculture and Technology, 3-5-8 Saiwai-cho, Fuchu, Tokyo 183-8509, Japan; johnmaninang3@yahoo.com (J.S.M.); yfujii@cc.tuat.ac.jp (Y.F.); 3 Center for Global Communication Strategies (CGCS), College of Arts and Sciences, The University of Tokyo, 3-8-1 Komaba, Meguro-ku, Tokyo 153-8902, Japan; 4 Department of Science of Biological Production, Tokyo University of Agriculture and Technology, 3-5-8 Saiwai-cho, Fuchu, Tokyo 183-8509, Japan; ssakae@cc.tuat.ac.jp

**Keywords:** functional properties, yam, *Dioscorea* spp., flour, drying methods

## Abstract

Research on the processing of yam (*Dioscorea* spp.) into flour is aimed at optimizing techniques to obtain a material with high physicochemical and functional properties. The present study investigates the effect of the processing techniques on the levels of inulin, organic acids, total phenolics (TP), antioxidant capacity, and polyphenol oxidase (PPO) and peroxidase (POD) activities, as well as on the physicochemical properties of flour derived from two yam species—*Dioscorea esculenta* and *Dioscorea bulbifera*. All tubers were peeled and sliced, then subjected to different processing techniques through blanching, soaking, and drying. The results revealed that freeze-drying appears to be the best technique in achieving the highest whiteness index of yam flour. This coincided well with the low phenolics content and POD activity, which suggests a reduced enzymatic browning reaction in the freeze-dried yam flour. On the other hand, chemical analyses showed that *D. esculenta* and *D. bulbifera* flours have the highest levels of inulin (23.0 and 27.8 g/100 g DW, respectively) and succinic acid (7.96 and 7.65 g/100 g DW, respectively) in the samples subjected to direct oven-drying. Oven drying without pretreatment neither blanching nor water steeping maintained antioxidant activity in the flour derived from both *D. esculenta* and *D. bulbifera*.

## 1. Introduction

Indonesia has a wide variety of carbohydrate-rich foods, among which is yam tuber (*Dioscorea* spp.). Research has shown that yam tubers, particularly *Dioscorea esculenta* and *Dioscorea bulbifera*, are rich in inulin, organic acids, phenolics, and other antioxidants [1,2,3,4]. Inulin is popularly used as an ingredient of low fat products [5], is known to improve gastric health [6], prevent diabetes and carcinoma [7,8,9], and be immunomodulatory agent [10]. While, organic acids (i.e., oxalic acid, citric acid and succinic acid) are important constituents influencing yam’s organoleptic characteristics and are essential for human metabolism [11,12]. Antioxidant refers to a compound that can delay or inhibit the oxidation of lipids or other molecules by inhibiting the initiation or propagation of oxidative chain reactions and which can thus prevent or repair damage done to the body’s cells by oxygen. It acts by one or more of the following mechanisms: reducing activity, free radical-scavenging, potential complexing of pro-oxidant metals and quenching of singlet oxygen [13]. These findings underscore the potential use of yam tubers not only as an energy source, but as a component of a disease-preventive diet [14].

One way of utilizing yam tubers is by processing them into flour, which is used as a substitute for wheat flour. In contrast to the perishable tuber, yam flour is an ideal raw material input for food applications [15] because of its longer shelf life and reduced bulk for convenience during transport and storage. Processing of tubers into flour generally involves peeling, slicing, blanching, drying, and milling by common practice. Drying, either by oven or freeze drying, is done to lower the moisture content, which extends the shelf life of the product. Other advantages of drying have been reported by Calín-Sánchez et al. [16]. Oven-drying (convective drying) is commonly used for its simple design, easy operation, and low cost. On the other hand, freeze-drying helps prevent oxidation damages, minimize degradation of chemical compounds, reduces shrinkage and shift of soluble solids, preserve volatile compounds, and maintain the porous structure.

Processing techniques also trigger changes in the bioactive components of yam flour. Oven-drying using hot air at low temperature has been reported to increase total soluble polyphenol content, stronger DPPH scavenging activity and reducing power of yam flour [17]. Akissoe et al. [18] reported that steeping in water for an hour before the blanching step also reduces POD activity [18]. While, blanching decreases the activities of polyphenol oxidase (PPO) and peroxidase (POD) that catalyze the oxidation reaction leading to undesirable browning of the flour [19]. These processing techniques, thus appear to improve the attributes of the resulting yam flour from the perspective of product quality and health.

A number of studies have linked the steps and conditions involved in the processing on the quality attributes of yam flour [19,20]. However, their influence on the health-beneficial components (i.e., inulin, organic acids and total antioxidant activity) of the flour needs to be further understood. The present study aims to investigate the influence of different processing techniques on the functional, as well as the physicochemical, properties of yam flour.

## 2. Materials and Methods

### 2.1. Ingredients

Two yam species (*D. esculenta* and *D. bulbifera*) from Yogyakarta, Indonesia, were washed, peeled, and sliced (2 mm thickness). The lot was then portioned into four treatment groups as described in Table 1. Oven-drying at 60 °C was based on the method of Hsu et al. [21] and was accomplished using a convection oven (MOV-112F(U), SANYO Electric Biomedical Co., Ltd., Osaka, Japan) while freeze-drying was done in an FD-80 freeze dryer (EYELA, Tokyo Rikakikai Co. Ltd., Tokyo, Japan). The conditions for the pretreatment step of blanching were as reported by Akissoe et al. [19] using a thermostat-controlled water bath, while those for the water steeping pretreatment were as described by Akissoe et al. [18].

The dried slices were then milled using a high-speed blender (Model WB-1, Osaka, Japan) and sieved through a 60-mesh screen. The yam flour was sealed in polyethylene laminated bags to prevent moisture absorption and stored in a freezer (−20 °C) until used for further analysis.

### 2.2. Physicochemical Properties of Yam Flour

Moisture contents of yam flour were measured by the air oven method of the Association of Official Analytical Chemists (AOAC) [22]. The reported values are means of triplicate samples with standard deviation. Percent yield (% *w/w*) was calculated by weighing the yam flour, which was produced after drying. Color profiles (Hunter L, a, and b coordinates) were measured using a chromameter (Minolta, CR-200, Osaka, Japan). These values were used to calculate the whiteness index (WI), commonly used to indicate the color quality of flour, with the equation.

(1) $$\mathrm{WI}=100-\sqrt{{\left(100-L\right)}^{2}+a^{2}+b^{2}}$$

where L, a, and b are the hunter L, a, and b values.

### 2.3. Total Phenolics

Total phenolics (TP) were measured according to previously described methods by Hung et al. [23] and Okarter [24] with results expressed using gallic acid (GA) as external standard (mg GA Equivalent g^−1^ DW). Yam flour (1 g) were mixed with 10 mL of 80% chilled ethanol (Kanto Chemical Inc., Tokyo, Japan) for 20 min with continuous shaking at 27 °C. The suspension was centrifuged and the supernatant was collected. The residue was re-extracted twice with 10 mL of 80% chilled ethanol and all supernatants were combined. The phenolic extracts were concentrated using a rotary evaporator (Eyela, Tokyo, Japan) and then filled up with methanol to a final volume of 10 mL. The phenolic compounds were then stored at −20 °C until use.

The phenolic contents in yam flour were determined using Folin-Ciocalteu’s method. The phenolic extracts were diluted to an appropriate concentration. The diluted solution (0.5 mL) was then oxidized with Folin-Ciocalteu’s reagent (0.5 mL, Wako Pure Chemical Industries Ltd., Osaka, Japan) and then neutralized with saturated sodium carbonate solution (1 mL, Wako Pure Chemical Industries Ltd., Osaka, Japan). The volume was adjusted to 10 mL with distilled water, then thoroughly mixed and allowed to stand for 45 min at ambient temperature. The solution was centrifuged for 5 min at 4000× *g* and the absorbance of the clear supernatants was measured at 725 nm using a spectrophotometer (GeneQuant 1300, Fisher Scientific, Loughborough, UK). A standard calibration was prepared using gallic acid (Nacalai Tesque, Kyoto, Japan) and the content of total phenolics in each extract was calculated and expressed as milligrams of gallic acid equivalent (GAE) per gram of the sample.

### 2.4. Polyphenol Oxidase Activities

Polyphenol oxidase (PPO) activities were determined spectrophotometrically as described previously by Bach et al. [25] with minor modifications and expressed as ΔA450 nm min^−1^ g^−1^ DW. Enzyme extracts were prepared from 5 g yam flour by homogenization of 25 mL cold extraction solution (0.05 M potassium phosphate buffer; pH 6.3; 0.15 M NaCl). The homogenate was held at 5 °C for 2 h, then centrifuge (high-speed refrigerator centrifuge CR22N, Hitachi, Japan) at 32,300× *g* at 4 °C for 10 min, and filtered through a 0.45 µm filter (Minisart, Sartorius stedim, Gottingen, Germany). The filtrate was then kept in an ice bath. The PPO assay was performed using caffeic acid (Nacalai Tesque, Kyoto, Japan) as substrate. A total of 0.5 mL of substrate (1 mg/mL caffeic acid) was mixed with 0.6 mL of filtrate and the absorbance was measured at 450 (A450) using a spectrophotometer (GeneQuant 1300, Fisher Scientific, Loughborough, UK). The sample A450 was recorded at 1 min intervals for 7 min vs. a 1.1 mL blank containing no substrate. The net A450 was tabulated and plotted against time and the slope was calculated for the linear portion of the curve.

### 2.5. Peroxidase Activities

Peroxidase (POD) activity was determined with a procedure adapted from previously reported methods by Akissoe et al. [18] and Mestres et al. [26]. Peroxidase was extracted from 50 mg of yam flour with 1 mL of 0.2 M potassium phosphate buffer (pH 7.0) for 15 min at ambient temperature followed by centrifugation (High-speed refrigerator centrifuge CR22N, Hitachi, Japan) at 7000× *g* for 5 min. The substrate was prepared just before use by mixing 0.5 mL of 1% (p/v) aqueous pyrogallol (Wako Pure Chemical Industries Ltd., Osaka, Japan) with 6 mL of 0.3% (*v/v*) aqueous H_2_O_2_ (Wako Pure Chemical Industries Ltd., Osaka, Japan). The reaction was run in 1.5 mL UV-cuvette semi-micro containing 0.05 mL of peroxidase extract and 1.45 mL of substrate; the blank was obtained by replacing the peroxidase extract by pure buffer (pH 7.0). The maximum increase in absorbance at 460 nm was determined; one unit of peroxidase activity was defined as an increase in absorbance of 0.001 min^−1^.

### 2.6. DPPH Radical Scavenging Capacities

The scavenging of the stable DPPH radical was widely used to evaluate antioxidant activity of phenolic compounds extracted from tuber, cereal, and fruit [1,21,23,27]. It was based on the measurement of the reducing ability of antioxidants toward DPPH [28]. DPPH radical scavenging capacities of yam flour were determined according to the method described by Hung et al. [23] and Huang et al. [28]. The concentration of DPPH (1.1-diphenyl-2-picrylhydrazyl, Tokyo Chemical Industry Ltd., Tokyo, Japan) solution used was 0.075 mM. The phenolic extracts (0.1 mL) were mixed with DPPH solution (3.9 mL), kept in the dark at ambient temperature, and the absorbance of the mixtures was recorded at 515 nm using spectrophotometer (GeneQuant 1300, Fisher Scientific, Loughborough, UK) for exactly 30 min. Blank was made from 3.9 mL of DPPH and 0.1 mL methanol, and measured the absorbance at t = 0. The scavenging of DPPH was calculated according to the following equation [29].

(2) $$\%\;\mathrm{DPPH}\;\mathrm{scavenging}=\frac{\left(\mathrm{Abs}\;\left(t=0\right)-\mathrm{Abs}\;\left(t=30\right)\right)}{\mathrm{Abs}\;\left(t=0\right)}\times 100$$

where Abs(t = 0) represents the absorbance of DPPH radical + methanol at t = 0 min, and Abs(t = 30) is the absorbance of DPPH radical + phenolic extracts at t = 30 min.

### 2.7. Inulin

The inulin in yam flour was determined using an adjusted procedure as described previously by Takeuchi et al. [3]. Soluble materials were extracted by incubating 1.0 g of the yam flour with 15 mL distilled water for 20 min at 80 °C. Then, the suspension was centrifuged (High-speed refrigerator centrifuge CR22N, Hitachi, Japan) for 5 min at 3000 rpm followed by filtration and the supernatant was recovered. Distilled water was added to the residue and the extraction procedure was repeated. The two supernatant fractions were combined and made up to a volume of 50 mL. Inulin was quantified by using HPLC. Inulin extract was filtered through membranes of 2 units of C18 cartridges (Sep-Pak, Waters, Ireland), 1 unit of CM cartridges (Sep-Pak, Waters, Ireland), and 1 unit of QMA cartridges (Sep-Pak, Waters, Ireland) in a *sequential arrangement* and stored at −30 °C until use. Inulin was measured by HPLC (Shimadzu, Kyoto, Japan) equipped with a Shim-pack SCR-101N column (Shimadzu, Kyoto, Japan) and refractive index detector RID-10A (Shimadzu, Kyoto, Japan). Column oven temperature was 80 °C, flow rate was 0.25 mL/min with a pump, and pure water was used as the carrier. Inulin (Wako Pure Chemical, Osaka, Japan) was used as standards.

### 2.8. Organic Acids

The organic acids were determined by HPLC, using the extraction and analysis method, as described previously by Bhandari et al. [1]. A total of 1 g of yam flour was added to 25 mL of distilled water, and 1 mL of the internal standard (10 g of glutaric acid (Tokyo Kasei Kogyo Co. Ltd., Tokyo, Japan) in 100 mL of water) was added. The mixture was placed in a boiling water bath for 10 min, cooled, and made up to volume in a 100 mL standard flash. A small volume of solution was filtered through a no. 5 A filter paper (Advantec Toyo Roshi Kaisha, Tokyo, Japan), and this solution was then again filtered through a 0.45 µm before separation by HPLC. A 7.8 × 300 mm ion exclusion column (HPX-87H, Bio-Rad, Hercules, CA, USA) was used with 0.0125 M H_2_SO_4_ as mobile phase, at a flow rate of 0.5 mL/min, the Diode Array Detector (DAD) operating at 214 nm and column oven temperature was 30 °C. For calibration of the HPLC system, a standard mixture of oxalic acid (0.02 g, Wako Pure Chemical Industries Ltd., Osaka, Japan), citric acid (0.02 g, Wako Pure Chemical Industries Ltd., Osaka, Japan), succinic acid (0.02 g, Wako Pure Chemical Industries Ltd., Osaka, Japan), and glutaric acid (0.08 g, Tokyo Kasei Kogyo Co. Ltd., Tokyo, Japan) in 100 mL of 0.0125 M H_2_SO_4_ was used.

### 2.9. Statistical Analysis

The experimental used completely randomized design with three replications. Normally distributed data with homogenous variance were subjected to analysis of variance (ANOVA) and DMRT test was used for comparison of the means, utilizing SAS software (Statistical Analysis System for windows, 9.1, SAS Institute Inc., Cary, NC, USA). All other data were subjected to the Kruskal–Wallis rank sum test using Minitab 14.

## 3. Results and Discussion

### 3.1. Physicochemical Properties of Yam Flour

Yield of yam flour species *D. esculenta* and *D. bulbifera* ranged from 20.64–29.17% and 18.02–27.52%, respectively (Table 2). Water-steeped and blanched flour (SB-Oven) had the lowest yield, while the flour without pretreatment (Oven) had the highest yield regardless of species used. The lowered yield of the pretreated groups may have occurred due to leaching during steeping and blanching leading to a loss in total solid as previously reported by Xiao et al. [30].

The moisture content of yam flour from both species in all treatment groups were around 2.49 to 6.72% or less than 15% (Table 2), which is consistent with the safety requirement of the Codex Alimentarius Commission for wheat flour [31]. The low moisture content is necessary to avoid microbial contamination, thus prolonging the shelf life of the flour, and is convenient for long-distance distribution due to its low bulk and weight.

Enzymatic browning is the main problem in flour production due to the oxidative reaction occurring in various tubers. This lowers the physical quality attributes of the product, especially color. Color profiles of yam flour in this study were indicated by the L, a, b values, which were in turn used for calculating the whiteness index (WI).

Color of yam flour prepared with different processing techniques are shown in Table 3. The L-values of *D. bulbifera* flour were between 74.84 and 92.24. In order to prevent the discoloration during processing, yam slices were pretreated with blanching and steeping steps before drying. The yam flour from both species that were water-steeped, blanched, and freeze-dried (SB-Freeze) had a lighter color as indicated by higher L-values.

The water-steeped, blanched, and freeze-dried flour (SB-Freeze) from both species used showed the lowest redness (indicated by a-values) and lowest yellowness (indicated by b-values) compared to flour produced by other treatments. These low colors attributes reflected on the significant increase in whiteness index (WI) of this group. The SB-Freeze treatment had significant effect on increasing whiteness index of *D. esculenta* and *D. bulbifera* flour.

Color changes due to the drying process can affect the final food quality. Freeze-drying applied in this study prevents color degradation better than oven-drying. This method also produces a less damaging color than air-drying and spray-drying [32]. Freeze-drying, known as lyophilization, is a process involving a sublimation to remove water in the form of ice under low pressure from a material. This process is the best food dehydration technique in many applications to produce high-quality food and pharmaceuticals [33].

### 3.2. Functional Properties of Yam Flour

Enzymatic browning is linked to polyphenol oxidase (PPO) and peroxidase (POD) activities, and the production of polyphenols and their derivates [34,35,36]. These enzymes, the PPO and POD, catalyze the oxidation of phenolic substrate with different ways, promote deteriorate reactions and consequent undesirable changes in nutritional value, flavor, and color (including dark pigments) [37,38]. In general, the browning reaction can affect the food quality of flour, in a negative manner. The treatments of combination of steeping and blanching and freeze-drying (SB-freeze) seem to have a significant effect on decreasing of PPO and TP of *D. esculenta* flour. Table 4 shows the blanching can reduce PPO, which is known as one of the effective interventions that can inhibit this parameter activities as reported by Shrestha et al. [39]. The PPO and TP of this yam flour are between 0.02 and 1.00 ΔA450 nm min^−1^ g^−1^ DW and 3.19 and 11.86 mg GAE/g, respectively. The loss of TP content was caused by leaching during the blanching. This phenomenon may be explained by the fact that phenolics are highly soluble in water [40,41]. The result is in agreement with a previous study reported by Xiao et al. [30]. The reduction of TP in several vegetables due to boiling and blanching processes has also been previously reported by some researchers [42,43,44,45].

On the contrary, B-Oven, SB-Oven, and SB-freeze treatments resulted in little effect on the PPO activity and TP of *D. bulbifera* flour (Table 4). The PPO and TP of *D. bulbifera* flours are between 0.04 and 0.37 ΔA450 nm min^−1^ g^−1^ DW, and between 4.17 and 7.95 mg GAE/g, respectively. Several phenolic compounds, including ferulic acid, catechin, and tannin found in *Dioscorea* spp., have been reported by Akissoe et al. [19] and Polycarp et al. [46]. Ferulic acid, a derivative of cinnamic acid, was identified with concentrations ranging from 0.03 to 0.04 µM.g-1. Catechin, representative of flavanols was also identified with concentrations ranging from 0.26 to 0.41 µM g^−1^. Low level of tannin was found with concentration < 15 mg/100 g. Phenolic compounds are a main class of secondary metabolites in fruit and vegetable, and are divided into phenolic acids and polyphenols [38].

The POD activities of *D. esculenta* and *D. bulbifera* flour produced by different processing techniques are between 2249.78 and 7228.48 unit g^−1^ DW. The treatments involved in the processing seem to have no significant effect on POD of *D. esculenta* flour. While, POD of *D. bulbifera* flour significantly increased during freeze-drying.

Contrary to the previous report [47], the results showed POD activities in all yam species cannot be inactivated with blanching and steeping pretreatments. Freeze-drying seems to be ineffective in keeping the POD enzyme activity low in both *D. esculenta* and *D. bulbifera* flour. This, however did not reflect in the whiteness index of the flour from this treatment group since our samples were kept in the freezer (−20 °C) prior to analysis. However, this implicates a probably rapid color degradation of this flour during storage in the conditions commonly used by the industry [48].

The percentage of DPPH scavenging of yam flour are between 5.00 and 18.24% (Table 4). The result showed that pretreatment and different drying techniques seemed not to have a significant effect on reducing DPPH scavenging capacity in yam flour. Both *D. esculenta* and *D. bulbifera* flour from treatment group of Oven (control) have the highest DPPH scavenging capacities due to the higher TP content. The lower TP content in samples groups of B-Oven, SB-Oven, and SB-Freeze was caused by leaching during steeping and blanching, consistent with the previous report of Xiao et al. [30]

Inulin in *D. esculenta* and *D. bulbifera* flour range from 4.99 to 27.80 g/100 g DW (Table 5). In this study, the amount of inulin in both yam flour seems to be not significantly affected by pretreatment and drying process. Nonetheless, the result was consistent with the previous report [3], yam flour produced by process of Oven (control) had the highest retention of the inulin content of the raw material compared to the other groups with pretreatment steps. In this study, the result showed that direct oven-drying technique may be the alternative technique to retain inulin content in yam flour. In conclusion, the blanching and soaking were not recommended for producing inulin powder. A previous study reported by Zhu et al. [49] showed that a production process using spray-drying and freeze-drying without pretreatment can result in yield of inulin powder that reached 8.65 and 7.02%, respectively.

For the different application, oven-drying can also be used as a pretreatment prior to extraction and spray drying process as developed in the previous study [50] to produce maximum inulin powder yield from *Dioscorea* spp. The spray drying at 119.20 °C, the creeping speed of 21.64 rpm, and the applied pressure of 0.03 MPa resulted in 11.96% of inulin product yield from chicory (*Cichorium intybus* L.).

Succinic acid appears to be the prominent organic acid in both species of yam. Succinic acid concentrations in *D. esculenta* and *D. bulbifera* flour ranged between 549.7 and 7957.7, and 447.8 and 7653.7 mg/100 g DW, respectively. Citric acid was determined to be the second most abundant organic acid in yam flour. Citric acid concentrations in *D. esculenta* and *D. bulbifera* flour ranged between 47.5 and 1739.2, and 30.2 and 1374.1 mg/100 g DW, respectively. Oxalic acid was determined in considerable amount in all yam species. The concentration of oxalic acid in *D. esculenta* and *D. bulbifera* flour were found to be 11.8 and 31.2, and 11.8 and 18.8 mg/100 g DW, respectively.

Blanching, steeping and freeze-drying significantly decreased the amount of succinic acid in the yam flour of both *D. esculenta* and *D. bulbifera*. While, decreasing of oxalic acid is apparently affected by the blanching and drying steps in *D. esculenta* flour but not in *D. bulbifera* flour. Oven drying without pretreatment significantly maintained the amount of organic acid in all yam species. Table 5 shows the variation in concentrations of individual organic acid in different yam species. These results are in agreement with that of the previous study [1]. The organic acid composition of fruits and vegetables is not easily predictable and is largely dependent on growing regions, climates, and the varieties [51].

Organic acids as phenolic compounds have a preventive role against various diseases due to their antioxidant properties. Oxalic, citric, and succinic acids are able to chelate metals [52]. Citric acid has responsibility in bone health and it is involved in bone metabolism [53]. Succinic acid contributes to helping with diabetes treatment [12].

### 3.3. Correlation between Color, Functional Properties, and Antioxidant Activities

Correlations between TP content or PPO activities or POD activities and color (L-values and WI) are given in Table 6. There were strong negative correlations between TP content or PPO activities and L-values or whiteness index in *D. bulbifera* flour. A previous study [54] showed that browning index is proportionally correlated with the TP content of yam flour. Consistently, the lightness of shade (L-value) and WI of *D bulbifera* flour increased with decreasing TP content and PPO activity.

In contrast, a positive correlation was seen between POD activities and color (L-values and WI) in *D. bulbifera* flour. These results show that browning index did not correlate with POD activity of *D. bulbifera* flour.

A strong positive correlation was shown between TP content and antioxidant activity in *D. esculenta* flour (r = 0.931 ****). Antioxidant activity of the yam flour increased with increasing its TP content. In food, polyphenols may contribute to the bitterness, astringency, color, flavor, odor, and oxidative stability [55]. Polyphenols are the most abundant antioxidants in human diet. As antioxidants, polyphenols may protect cell constituents against oxidative damage and, therefore, limit the risk of various degenerative diseases associated to oxidative stress [56].

## 4. Conclusions

Functional properties of yam flour prepared by different processing techniques have been determined. Inulin, total phenolic content and succinic acid were highly preserved by direct oven drying without pretreatment. The addition of steeping and blanching steps as pretreatment to drying led to considerable losses of the functional constituents of yam. However, flour prepared without pretreatment displayed massive discoloration indicating the importance of optimization in the preparation processing of yam flour. Antioxidant activity in *D. esculenta* flour increased with increasing total phenolics content. Freeze-drying in the production of *D. bulbifera* flour either improved physical properties or reduced enzymatic browning. Enzymatic browning was due to the production of polyphenols and PPO activity, but not POD activity. Antioxidant activity in *D. esculenta* flour increased with increasing of TP content. With further research, these results may prove valuable in customizing the processing steps and conditions to achieve the desired functional characteristics of yam flour intended for the production of foods and nutrition with special dietary uses. Yam flour as an intermediate product has health-promoting characteristics that can be developed in many sectors, responding to the worldwide trend toward foods that have nutrition and health benefits.

## Figures and Tables

**Table 1:** Description of the post-slicing steps and conditions of each treatment group in the processing of yam flour.

Treatment	Description
Oven	Yam slices were only oven-dried at 60 °C until the weight stabilized
B-Oven	Yam slices were blanched (70 °C, 10 min) and oven-dried as Oven
SB-Oven	Yam slices were steeped in water (28–30 °C, 1 h) then blanched and oven-dried as B-Oven
SB-Freeze	Yam slices were treated as SB-Oven except the drying was by freeze-dryer

**Table 2:** Yield and moisture content of yam flour prepared with different processing techniques.

Yam Species/Treatment	Moisture Content (%)	Yield (%)
*D. esculenta*		
Oven	5.62 ± 0.16	29.17 ± 0.30
B-Oven	5.92 ± 0.19	25.28 ± 0.52
SB-Oven	5.22 ± 0.18	20.64 ± 0.31
SB-Freeze	3.46 ± 0.11	22.24 ± 0.44
*D. bulbifera*		
Oven	6.72 ± 0.19	27.52 ± 1.65
B-Oven	5.78 ± 0.27	22.46 ± 0.36
SB-Oven	5.67 ± 0.03	18.02 ± 2.23
SB-Freeze	2.49 ± 0.13	21.31 ± 0.14

Each value is expressed as the means ± SD (*n* = 3). Treatment groups: Oven, oven-dried only; B-Oven, blanched at 70 °C for 10 min and oven-dried; SB-Oven, water-steeped at 28–30 °C for 1 h) then blanched and oven-dried; SB-Freeze, pretreated as SB-Oven, except slices were freeze-dried.

**Table 3:** Color of yam flour prepared with different processing techniques.

Yam Species/Treatment	Color Attributes			
	L-Value	a-Value	b-Value	Whiteness Index
*D. esculenta*				
Oven	79.80 **	0.74 ± 0.17 ^b^*	12.94 ± 0.13 ^ab^*	76.03 **
B-Oven	80.58 **	1.57 ± 0.69 ^a^*	14.44 ± 1.41 ^a^*	75.44 **
SB-Oven	83.37 **	0.92 ± 0.05 ^ab^*	11.84 ± 0.55 ^b^*	79.80 **
SB-Freeze	92.39 **	−0.27 ± 0.14 ^c^*	6.91 ± 0.31 ^c^*	89.91 **
*p*-Value	0.025			0.024
*D. bulbifera*				
Oven	74.84 ± 1.03 ^b^*	3.44 ± 0.32 ^a^*	18.46 **	68.84 ± 1.32 ^b^*
B-Oven	76.01 ± 4.02 ^b^*	2.62 ± 0.96 ^a^*	17.87 **	70.45 ± 4.66 ^b^*
SB-Oven	77.23 ± 4.04 ^b^*	2.75 ± 1.04 ^a^*	18.12 **	71.02 ± 4.54 ^b^*
SB-Freeze	92.24 ± 0.69 ^a^*	−0.18 ± 0.35 ^b^*	6.59 **	89.04 ± 1.80 ^a^*
*p*-Value			0.094 ***	

* value is expressed as the mean ± SD (*n* = 3). Different letters in the same column and species showed significant differences (*p* < 0.05) by DMRT test. ** value is expressed as the median. Letters followed by *** showed no significant different according to the Kruskal–Wallis test (*p* > 0.05). Treatment groups: Oven, oven-dried only; B-Oven, blanched at 70 °C for 10 min and oven-dried; SB-Oven, water-steeped at 28–30 °C for 1 h then blanched and oven-dried; SB-Freeze, pretreated as SB-Oven, except slices were freeze-dried.

**Table 4:** Functional properties of yam flour prepared with different processing techniques.

Treatment	PPO (ΔA450 nm min^−1^ g^−1^ DW)	TP (mg GAE/g)	POD (unit g^−1^ DW)	DPPH (%)
*D. esculenta*				
Oven	1.01 **	11.87 **	6958.25 **	14.92 **
B-Oven	0.14 **	4.39 **	2376.24 **	4.98 **
SB-Oven	0.30 **	3.25 **	3000.00 **	5.70 **
SB-Freeze	0.02 **	3.56 **	6336.63 **	4.18 **
*p*-value	0.016	0.024	0.094 ***	0.082 ***
*D. bulbifera*				
Oven	0.37 ± 0.18 ^a^*	7.95 ± 0.32 ^a^*	3311.70 ± 238.01 ^b^*	18.24 ± 3.45 ^a^*
B-Oven	0.31 ± 0.09 ^a^*	6.36 ± 0.76 ^ab^*	2249.78 ± 618.33 ^b^*	9.44 ± 2.15 ^a^*
SB-Oven	0.22 ± 0.02 ^ab^*	5.47 ± 0.57 ^bc^*	2652.09 ± 409.96 ^b^*	12.21 ± 2.44 ^a^*
SB-Freeze	0.04 ± 0.00 ^b^*	4.17 ± 1.31 ^c^*	5642.65 ± 1461.65 ^a^*	10.43 ± 8.04 ^a^*

* value is expressed as the mean ± SD (*n* = 3). Different letters in the same column and species showed significant differences (*p* < 0.05) by DMRT test. ** value is expressed as the median. Letters followed by *** showed no significant different according Kruskal-Wallis test (*p* > 0.05). Treatment groups: Oven, oven-dried only; B-Oven, blanched at 70 °C for 10 min and oven-dried; SB-Oven, water-steeped at 28–30 °C for 1 h then blanched and oven-dried; SB-Freeze, pretreated as SB-Oven, except slices were freeze-dried.

**Table 5:** Inulin and organic acids compositions of yam flour prepared with different processing techniques.

Yam Species/ Treatment	Inulin (g/100 g DW)	Organic Acids (mg/100 g DW)		
		Oxalic Acid	Citric Acid	Succinic Acid
*D. esculenta*				
Oven	22.58 **	31.16 ± 0.79 ^a^*	1632.86 **	7957.72 ± 147.76 ^a^*
B-Oven	4.13 **	11.79 ± 1.20 ^c^*	227.46 **	2760.39 ± 448.07 ^b^*
SB-Oven	7.24 **	12.74 ± 1.62 ^c^*	35.11 **	1262.96 ± 255.27 ^c^*
SB-Freeze	3.60 **	19.47 ± 2.00 ^b^*	49.86 **	549.68 ± 234.35 ^d^*
*p*-Value	0.066 ***		0.016	
*D. bulbifera*				
Oven	27.80 **	14.62 ± 2.39 ^a^*	1336.72 **	7653.66 ± 583.98 ^a^*
B-Oven	8.29 **	15.98 ± 6.08 ^a^*	154.17 **	5488.22 ± 450.02 ^b^*
SB-Oven	7.08 **	18.77 ± 1.77 ^a^*	33.37 **	2283.08 ± 502.47 ^c^*
SB-Freeze	7.13 **	11.81 ± 0.63 ^a^*	29.07 **	447.83 ± 164.99 ^d^*
*p* -Value	0.092 ***		0.024	

* value is expressed as the mean ± SD (*n* = 3). Different letters in the same column and species showed significant differences (*p* < 0.05) by DMRT test. ** value is expressed as the median. Letters that followed by *** showed no significant different according Kruskal-Wallis test (*p* > 0.05). Treatment groups: Oven, oven-dried only; B-Oven, blanched at 70 °C for 10 min and oven-dried; SB-Oven, water-steeped at 28–30 °C for 1 h then blanched and oven-dried; SB-Freeze, pretreated as SB-Oven, except slices were freeze-dried.

**Table 6:** The correlation coefficient (r) between parameters of yam flour.

Parameters	TP (mg GAE/g DW)	PPO (ΔA450 nm min^−1^ g^−1^ DW)	POD (unit g^−1^ DW)
*D. esculenta*			
Antioxidant Activities (% DPPH scavenging)	0.931 ****	ns	ns
*D. bulbifera*			
L	−0.754 **	−0.807 **	0.781 **
WI	−0.766 **	−0.802 **	0.804 **
TP (mg GAE/g DW)	1.00	ns	ns
PPO (ΔA450 nm min^−1^ g^−1^ DW)	ns	1.00	ns
POD (unit g^−1^ DW)	ns	ns	1.00

** showed significant correlation *p* < 0.01, **** showed significant correlation *p* < 0.0001, ns = not significant.
